# Associated factors with constipation and health-related quality of life in lung cancer patients with platinum-based chemotherapy: A cross-sectional study

**DOI:** 10.1097/MD.0000000000026547

**Published:** 2021-07-30

**Authors:** Huamao Chen, Xixi Gu, Yan Zhang, Jian Feng, Yan Gu

**Affiliations:** a Department of Respiratory, Affiliated Hospital of Nantong University; b Medical school, Nantong University, Nantong, Jiangsu Province, China.

**Keywords:** associated factors, constipation, lung cancer, platinum-based chemotherapy, quality of life

## Abstract

The main purpose of this study was to investigate current state of constipation for lung cancer (LC) patients receiving platinum-based chemotherapy. The relationships between social demography, clinical variables, psychological status, and constipation were analyzed. In addition, quality of life (QoL) in LC patients with constipation was also analyzed. One hundred LC patients participated in this cross-sectional study. Under the guidance of the researchers, Functional Living Index-Emesis, Piper Fatigue Scale, Patient Health Questionnaire, Generalized Anxiety Disorder-7, European Organization for Research and Treatment of Cancer (EORTC) QLQ-C30 (version 3.0), Pittsburgh Sleep Quality Index, General Well-being Scale, Social Support Rate Scale, General Self-Efficacy Scale, and other related questionnaires were completed. The result showed the symptom of constipation was observed in 41 (41%) LC patients. The occurrence and development of constipation were associated with gender, food intake, exercise, nausea, fatigue, anxiety, depression, sleep disorders, and happiness. The study also found patients with constipation had significant lower QoL scores, especially the score in the general state. Constipation was very common in LC patients undergoing platinum-based chemotherapy. Reduced food intake and fatigue were the independent factors. Constipation significantly affects the QoL of the patients. Therefore, more attention should be paid to the risk factors of constipation in LC patients undergoing platinum-based chemotherapy, the earlier intervention was done to these patients, the better to improve their QoL.

## 1 Introduction

Lung cancer (LC) is the most common malignancy in terms of both morbidity and mortality. It is the leading cause of death of all kinds of malignant tumors.^[1]^ Many symptoms of LC are not specific until late stages.^[2]^ The primary goal of systemic therapy in patients with advanced LC is to relieve symptoms and improve the quality of life and prolong the survival duration.^[3]^ Chemotherapy is one of the main treatment methods of intermediate and advanced LC. Platinum drugs are used to treat a wide variety of cancers, including LC.^[4]^ In terms of chemotherapy regimen selection, combination chemotherapy containing platinum is recommended as the first-line treatment.^[5]^ Although first-line platinum-based combination chemotherapy improves survival, palliates symptoms, and improves quality of life, it is accompanied by severe side effects such as loss of appetite, nausea, and vomiting.^[6]^ Constipation was one of the most common and painful side effects of cancer treatment.^[7]^

Constipation refers to the symptom of infrequent bowel movement that causes fewer stool passing and abdominal pain. Severe constipation can cause blockages in rectum and may lead to surgeries.^[8]^ It is a life-threatening disease.^[9]^ In addition, constipation also leads to increased incidence of coronary heart disease and ischemic stroke.^[10]^ It also causes severe psychological symptoms such as anxiety and depression.^[7]^ However, constipation is usually considered a private matter and rarely discussed by patients, so it is easy to be neglected.^[11]^

Constipation severely affects the patients’ quality of life. Treatment for constipation may lead to substantial healthcare resource utilization and healthcare costs. It may impose a considerable social and economic burden.^[12]^ Current studies were limited to the diagnosis, treatment, and clinical management of chronic constipation and opioid-induced constipation. Currently, influencing factors and quality of life of constipation for LC patients with platinum chemotherapy are rarely reported. Precise measures can be taken to prevent constipation if the influencing factors of constipation in LC patients with platinum chemotherapy were carefully analyzed. In this study, present situation of constipation, influencing factors, and the relationship between constipation and quality of life in LC patients with platinum chemotherapy were studied and discussed.

## 2 Materials and methods

### 2.1 Participants

The cross-sectional study was conducted in the Affiliated Hospital of Nantong University from September 2019 to February 2020. The LC patients were hospitalized in Respiratory Department. The diagnosis of lung cancer was confirmed by pathology reports. Constipation is characterized by any of the following:^[13]^ reduced bowel movement frequency; development or worsening of straining to pass bowel movements; a sense of incomplete rectal evacuation; harder stool consistency.

This study was approved by the Ethics Committee of the Affiliated Hospital of Nan tong University (ethics approval number: 2019-K053). Before completing the related questionnaires, all study participants signed the informed consent form, the medical assessments were recorded by researchers.

### 2.2 Inclusion criteria and exclusion criteria

Inclusion criteria: diagnosis of LC; intravenous chemotherapy medications were used for treatment, at least a complete cycle; no known cognitive deficits. Exclusion criteria: not receive intravenous chemotherapy; unconsciousness and difficulties in communication; not complete the first cycle of chemotherapy; diagnosed with intestinal diseases or irritable bowel syndrome; patients who are taking opiates for long time or taking other constipation-inducing medications.

### 2.3 Methods

One hundred LC patients with platinum-based chemotherapy were participated in this research. All patients completed a set of standardized self-report questionnaires as the followings.

Demographic variables are as the following: age, sex, Education, Employment, Marital status, Chronic disease, and medical insurance. Clinical data such as pathological type, performance status (PS), C-reactive protein, erythrocyte sedimentation rate, prophylactic are used of laxatives and chemotherapy regimens which were obtained by searching their electronic medical records.

Functional Living Index-Emesis:^[14]^ It was mainly used to assess the impact of acute nausea and vomiting and delaying nausea and vomiting within 48 hours on the patient's quality of life, which was required to be completed at 5 days after chemotherapy.^[15]^ Higher scores indicate worse QoL.

Piper Fatigue Scale:^[16]^ The Piper Fatigue Scale consisted of 22 numerical items that assess fatigue experienced by the patients. All items were coded on a 0 to 10 numeric scale and resolved into 4 dimensions of subjective fatigue: behavior/severity, affective meaning, sensory, and cognitive/mood. The higher scores, the more fatigued.

Patient Health Questionnaire (PHQ-9): The 9 questions in the PHQ-9 evaluate for the frequency of depressive symptoms using a Likert scale from 0 (Not at all) to 3 (Nearly Every Day), with a score of 3 indicating greatest symptom frequency.^[17]^ A score ranged from 0 to 4 in Patient Health Questionnaire 9 indicates no depression. A score ranged from 5 to 27 in Patient Health Questionnaire 9 indicates depression.^[18]^

Generalized Anxiety Disorder-7 (GAD-7), no anxiety (score 0–4), mild anxiety (score 5–9), moderate anxiety (score 10–15), and severe anxiety (score 15–21) can be determined.^[19]^

The QoL of LC patients have been scored through the validated questionnaire: the European Organization for Research and Treatment of Cancer (EORTC) QLQ-C30 (version 3.0). The EORTC-QLQC30 questionnaire has 4-point scales for the first 5 items. These were coded with the same response categories as items 6–28, namely “Not at all,” “A little,” “Quite a bit,” and “Very much,” and 7-point scales for the items 29 and 30, namely “Very poor”-to-“Excellent.” The evaluation of the items of the EORTC QLQ-C30 questionnaire was done through the following algorithm: raw score (RS): (I1+I2+…+In)/n; functional scale score: 1 – {(RS – 1)/range} × 100; symptom scale or general state score: {(RS – 1)/range} × 10.0. The QoL scale of the EORTC was scored from 0 to 100, where higher scores in general state and functional scales represent a higher functional level, while in the symptoms scale, the higher the score, the greater symptomatology.^[20]^

The Pittsburgh sleep quality index is a subjective self-rated sleep quality scale for patients, including subjective sleep quality, sleep time, sleep efficiency, sleep disorders, hypnotics, and daytime dysfunction.^[21]^ The higher the total score, the worse the sleep quality.^[22]^

The general well-being scale is a structured tool, which is developed for the national center for health statistics to assess participants’ statements of happiness. There are 33 items in this scale. The higher the score, the happier the patients. The retest reliability of this scale is 0.8.^[23]^

The Social Support Rate Scale contained 10 items consisting of 3 grades, with an aggregate score that ranged from 7 to 56. A higher score indicated higher levels of social support.^[24]^

The General Self-Efficacy Scale is a 10-item self-report scale, with higher scores indicating a stronger belief that one's own actions are responsible for successful outcomes.^[25]^

### 2.4 Statistical analysis

Data were analyzed by SPSS (version 20.0). The Shapiro–Wilk test was used to evaluate the normal distribution. Mean value was used to evaluate the conformity to the normal distribution (± Standard Deviation); otherwise, the median was used (IQR) or number (percentage) to describe statistics. The independent samples *t* test was used to evaluate the difference between the 2 groups in terms of numerical variables. We used chi-square tests to evaluate the differences in proportions. Multiple factors were using multivariable logistic regression analysis (*P* < .05).

## 3 Results

### 3.1 Sample characteristics

In this study, data were analyzed from 100 LC patients with platinum-based chemotherapy. Seventy-nine percent of the participants were men, 58% of the subjects were highly educated, and 98% of the participants were married. According to the definition of constipation, 41 patients were constipated. The incidence of constipation was 41%. Demographic characteristics of the study are described in Table 1.

**Table 1:** Differences of sociodemographic variables between LC patients with and without constipation.

Characteristic	Total (N = 100)	Constipation (N = 41)	Nonconstipation (N = 59)	*P*
Age^∗^	63.12 ± 8.01	63.12 ± 8.01	63.07 ± 8.38	.729
Gender, male^†^	79 (79)	28 (68.3)	51 (86.4)	.028
Educational level^†^				.928
Highly educated^†^	58 (58)	24 (58.5)	34 (57.6)	
Lowly educated^†^	42 (42)	17 (41.5)	25 (42.4)	
Profession, yes^†^	54 (54)	20 (48.8)	34 (57.6)	.383
Marital status^†^				.234
Married	98 (98)	41 (100)	57 (96.6)	
Others	2 (2)	0 (0)	2 (3.4)	
Pattern of payment^†^				.275
Medical insurance^†^	48 (48)	17 (41.5)	31 (52.5)	
Others	52 (52)	24 (58.5)	28 (47.5)	
Smoke, yes^†^	72 (72)	26 (63.4)	46 (78)	.111
Accompanied by other diseases, yes^†^	34 (34)	16 (39)	18 (30.5)	.377

### 3.2 Difference in patient-reported behavior and outcomes between the two groups

Comparison of socioeconomic status and clinical and psychological characteristics among patients with or without constipation is presented in Tables 1 and 2.

**Table 2:** Differences of clinical and psychological characteristics between LC patients with platinum-based chemotherapy with and without constipation.

Characteristic	Total (N = 100)	Constipation (N = 41)	Nonconstipation (N = 59)	*P*
Reduced food intake, yes^†^	47 (47)	28 (68.3)	19 (32.2)	<.001
Exercise reduced, yes^†^	59 (59)	14 (34.1)	45 (76.3)	<.001
Nausea, yes^†^	31 (31)	19 (46.3)	12 (20.3)	.006
Emesis, yes^†^	10 (10)	6 (14.6)	4 (6.8)	.198
Fatigue, yes^†^	63 (63)	37 (90.2)	26 (44.1)	<.001
Anxiety score^‡^	1 (0, 3.75)	2 (0, 6)	0 (0, 2)	.023
Depression score^‡^	2 (0, 6)	4 (1.5, 8)	1 (0, 4)	.003
Sleep disturbance, yes^†^	60 (60)	31 (75.6)	29 (49.2)	.008
SSRS^∗^	38.2 ± 5.16	37.76 ± 4.97	38.51 ± 5.31	.476
GSES^∗^	28.48 ± 6.76	24.49 ± 6.1	29.17 ± 7.15	.223
GWB^∗^	83.33 ± 15.22	78.88 ± 15.35	86.42 ± 14.47	.014
Diagnose^†^				.505
Squamous carcinoma	25 (25)	8 (19.5)	17 (29.3)	
Adenocarcinoma	51 (51)	22 (53.7)	29 (50)	
Small cell carcinoma	23 (23)	11 (26.8)	12 (20.7)	
PS^‡^	1 (0, 2)	1 (1, 2)	1 (0, 1)	.154
Chemotherapy regimens^†^				.86
Cisplatin	29 (29)	11 (26.8)	18 (30.5)	
Nedaplatin	56 (56)	23 (56.1)	33 (55.9)	
Carboplatin	15 (15)	7 (17.1)	8 (13.6)	
Days of chemotherapy^‡^	2 (1, 2)	2 (1, 2)	2 (1, 2)	.979
First chemotherapy, yes^†^	48 (48)	18 (43.8)	30 (50.8)	.49
Targeted therapy, yes^†^	13 (13)	5 (12.2)	8 (13.6)	.84
Surgery, yes^†^	32 (32)	16 (39)	16 (27.1)	.21
Radiotherapy, yes^†^	1 (1)	1 (2.4)	0 (0)	.23
Complication, yes^†^	69 (69)	29 (70.7)	40 (67.8)	.76
Know the illness, yes^†^	74 (74)	32 (78	42 (71.2)	.44
Prophylactic use of laxatives, yes^†^	50 (50)	16 (39)	34 (57.6)	.067
WBC^∗^	6.13 ± 2.28	6.23 ± 2.24	6.06 ± 2.32	.729
Hb^∗^	125.93 ± 17.20	122.37 ± 17.84	128.45 ± 16.41	.083
A/G^∗^	1.58 ± 0.31	1.58 ± 0.32	1.58 ± 0.30	.995
D-II^‡^	0.57 (0.25, 1.22)	0.53 (0.27, 1.2)	0.61 (0.23, 1.31)	.977
ESR^∗^	34.06 ± 31.56	46 ± 39.01	27.5 ± 25.38	.177
CRP^‡^	4.53 (2.27, 14.87)	5.14 (2.33, 18.1)	3.72 (2.05, 10.39)	.306
CEA^‡^	3.6 (2.12, 9.3)	3.52 (2.65, 14.39)	3.6 (2.1, 7.6)	.199

Significant difference of gender, food intake, exercise, nausea, fatigue, anxiety, depression, sleep disorders, and general well-being between the 2 groups were found (*P* < .05). There was no significant difference in chemotherapy regimen, disease awareness, prophylactic use of laxative drugs, and others (*P*>.05).

### 3.3 Reduced food intake and fatigue were the main risk factors of constipation

Binary logistic regression analysis was conducted while constipation is the dependent variable and gender, food intake, exercise, nausea, fatigue, anxiety, depression, sleep disorders, and happiness are independent variables. The results showed that reduced food intake and fatigue were the main factors that affect constipation (Table 3).

**Table 3:** Constipation regression analysis in LC patients with platinum-based chemotherapy.

Characteristic	Standard	*P* value	Exp (B)	(95% CI)
Reduced food intake	0.48	.015	3.2	(1.25, 8.20)
Fatigue	1.003	0	9.3	(2.86, 30.29)

### 3.4 Declined QoL in LC patients with platinum-based chemotherapy with constipation

Comparing the 2 groups, we found that the QoL of LC patients with symptom of constipation were significantly lower than that of non-Constipation patients. There were 3 dimensions in QLQ-C30. After analysis of the difference of these dimensions between the 2 groups, it was found that the general state scores of constipate LC patients were significantly lower than those of nonconstipated LC patients. The symptom score of constipated LC patients was significantly higher than that of LC patients without constipation (Table 4). From the correlation analysis, constipation was positively correlated with functional score and general state score, but negatively correlated with symptom score (Table 5).

**Table 4:** Differences of quality of life between LC patients with platinum-based chemotherapy with constipation according to EORTC-QLQC30.

Characteristic	Total (N = 100)	Constipation (N = 41)	Nonconstipation (N = 59)	*P*
Functional scale^†^	447.5 (402.5, 484.58)	453.33 (388.33, 478.33)	446.67 (408.33, 486.67)	.394
Symptoms scale^∗^	144.83 ± 104.80	171.54 ± 108.8	126.27 ± 98.64	.033
General state^†^	66.67 (50, 83.33)	66.67 (50, 70.83)	75 (66.67, 83.33)	.009

**Table 5:** Correlations between constipation and QoL in LC patients with platinum-based chemotherapy.

QLQ-C30	Constipation r	*P*
General state	0.251	.012
Functional scale	0.069	.495
Symptoms scale	−0.214	.033

## 4 Discussion

Constipation is one of the most common complications for lung cancer who received chemotherapy. Once constipation occurs, it will significantly impact patients’ physical and mental health and consequently affect patients’ quality of life. However, constipation for lung cancer with platinum-based chemotherapy is often neglected by clinical workers. The estimated average incidence of constipation in adults was reported as high as 16% worldwide (varies between 0.7% and 79%).^[8]^ In this cross-sectional study, the incidence of constipation was 41%.

It has been reported that factors such as age, gender, and educational level influence the occurrence of constipation. Forootan et al reported that the incidence of constipation increases with age. Compared to men, women are more susceptible to constipation than men. Also, higher incidence of constipation is positively correlated with lower educational level.^[8]^ It was found that female LC patients receiving platinum chemotherapy are more likely to be constipated. Age and educational level were not to be found to be related to constipation in this study. Mugie et al also reported that women are more susceptible to constipation than men.^[26]^

The most common adverse reactions for platinum-based chemotherapy are nausea and vomiting, which leads to loss of appetite and reduced food intake. It was shown that reduced food intake is an independent factor for the constipation of lung cancer with platinum-type chemotherapy. In clinical treatment, more attention should be paid on preventing constipation in these patients.

Among the platinum-based chemotherapy regiments for LC, including cisplatin, carboplatin, nedaplatin, etc. Mitani et al have shown that the side effects of cisplatin are the greatest in platinum-based chemotherapy, especially on the occurrence of venous thromboembolic events.^[27]^ However, in this study, there was no statistical difference between different platinum-based chemotherapy on the occurrence of constipation (*P* > .05).

In clinical practice, prophylactic laxative drugs sometimes were used to prevent post-chemotherapy constipation. However, it was found that prophylactic use of laxative drugs has no preventive effect on the occurrence of constipation for LC patients with platinum-based chemotherapy, which has certain guiding significance for clinical treatment in this study. Also, there was a statistical difference between the 2 groups of sleep disorders, anxiety and depression (*P* < .05), which was consistent with relevant studies.^[28,29]^

Lung cancer and chemotherapy can also cause fatigue, Ghoshal et al have shown that constipation was relieved after the fatigue was improved.^[30]^ In this study, fatigue of LC patients with platinum-based chemotherapy is evidently observed and it was a predictive factor of constipation (*P* < .05). More attention needs to be paid on these patients.

Sumida et al have shown that the quality of life of constipated patients is significantly lower than that of nonconstipated patients.^[31]^ In this study, it was also found that QoL of LC patients with constipation was significantly lower than nonconstipation patients, especially the score in the dimension of general state.

There were several limitations of this study. First, this study was a cross-sectional study, which did not allow for following up the progress of the examined variables during the disease. Second, this study was conducted in a single hospital and the scales were all self-administered, which may lead to deviated results. Nonetheless, this research may still provide some valuable insights into this complex process by identifying the associations between variables and constipation of LC patients with platinum-based chemotherapy.

## 5 Conclusion

This is the first cross-sectional study to assess the situation of constipation in LC patients with platinum-based chemotherapy and explore the QoL of constipation patients.

Compared with other studies, variables are wider and systematic. From this study, a difference was found in food intake, exercise, nausea, fatigue, anxiety, depression, sleep disorders, and general well-being between the 2 groups. Besides that, patients with constipation had a considerable impaired QoL in comparison with nonconstipation. In the future, QoL of LC patients with constipation should be paid attention to by medical staff, which has important implications for improving disease management and treatment decisions. The small sample size and lead-time bias are limitations in this study. A larger sample, multicenter studies will be conducted to explore the factors that influence constipation with chemotherapy in the future.

## Acknowledgments

Huamao Chen would like to thank all my professors who have helped me to develop the fundamental and essential academic competence.

## Author contributions

Huamao Chen, Xixi Gu, Jian Feng and Yan Gu designed the study; Huamao Chen performed the research. Huamao Chen, Xixi Gu and Yan Zhan analyzed the data. Huamao Chen and Xixi Gu generated the figures and wrote the paper, revised the figures and critically revised the manuscript for important intellectual content. Yan Gu was responsible for quality control of the study, Huamao Chen and Xixi Gu contributed equally to this work.

**Conceptualization:** Huamao Chen, Xixi Gu, Jian Feng, Yan Gu.

**Data curation:** Huamao Chen, Xixi Gu.

**Formal analysis:** Huamao Chen, Xixi Gu.

**Methodology:** Jian Feng, Yan Gu, Huamao Chen.

**Software:** Huamao Chen, Xixi Gu, Yan Zhan.

**Writing – original draft:** Huamao Chen, Xixi Gu.

## References

[1] Kandathil A Iii RCS Subramaniam RM. Lung cancer recurrence: (18)F-FDG PET/CT in clinical practice. AJR Am J Roentgenology 2019;01–9. 10.2214/AJR.19.21227 31361525

[2] Tian Y Li M Song W. Effects of probiotics on chemotherapy in patients with lung cancer. Oncol Lett 2019;17:2836–48. 30854059 10.3892/ol.2019.9906 PMC6365978

[3] Arbour KC Riely GJ. Systemic therapy for locally advanced and metastatic non-small cell lung cancer: a review. JAMA 2019;322:764–74. 31454018 10.1001/jama.2019.11058

[4] Rossi A Di Maio M. Platinum-based chemotherapy in advanced non-small-cell lung cancer: optimal number of treatment cycles. Expert Rev Anticancer Ther 2016;16:653–60. 27010977 10.1586/14737140.2016.1170596

[5] Cao A He H Wang Q. Evidence of Astragalus injection combined platinum-based chemotherapy in advanced nonsmall cell lung cancer patients: a systematic review and meta-analysis. Medicine 2019;98:e14798. 30882655 10.1097/MD.0000000000014798 PMC6426520

[6] Socinski MA Schell MJ Bakri K. Second-line, low-dose, weekly paclitaxel in patients with stage IIIB/IV nonsmall cell lung carcinoma who fail first-line chemotherapy with carboplatin plus paclitaxel. Cancer 2002;95:1265–73. 12216094 10.1002/cncr.10835

[7] Shin J Park H. Effects of auricular acupressure on constipation in patients with breast cancer receiving chemotherapy: a randomized control trial. West J Nurs Res 2018;40:67–83. 27903827 10.1177/0193945916680362

[8] Forootan M Bagheri N Darvishi M. Chronic constipation: a review of literature. Medicine 2018;97:e10631. 29768326 10.1097/MD.0000000000010631 PMC5976340

[9] De Giorgio R Ruggeri E Stanghellini V. Chronic constipation in the elderly: a primer for the gastroenterologist. BMC Gastroenterol 2015;15:130. 26467668 10.1186/s12876-015-0366-3 PMC4604730

[10] Sumida K Molnar MZ Potukuchi PK. Constipation and risk of death and cardiovascular events. Atherosclerosis 2019;281:114–20. 30658186 10.1016/j.atherosclerosis.2018.12.021 PMC6399019

[11] Meinds RJ van Meegdenburg MM Trzpis M. On the prevalence of constipation and fecal incontinence, and their co-occurrence, in the Netherlands. Int J Colorectal Dis 2017;32:475–83. 27913883 10.1007/s00384-016-2722-3 PMC5355501

[12] Guerin A Carson RT Lewis B. The economic burden of treatment failure amongst patients with irritable bowel syndrome with constipation or chronic constipation: a retrospective analysis of a Medicaid population. J Med Econ 2014;17:577–86. 24811855 10.3111/13696998.2014.919926

[13] Camilleri M Drossman DA Becker G. Emerging treatments in neurogastroenterology: a multidisciplinary working group consensus statement on opioid-induced constipation. Neurogastroenterol Motility 2014;26:1386–95. 10.1111/nmo.12417 PMC4358801 25164154

[14] Helou J Thibault I Chu W. Quality of life changes after stereotactic ablative radiotherapy for liver metastases: a prospective cohort analysis. Radiotherapy Oncol 2018;129:435–40. 10.1016/j.radonc.2018.09.011 30274721

[15] Martin AR Carides AD Pearson JD. Functional relevance of antiemetic control. Experience using the FLIE questionnaire in a randomised study of the NK-1 antagonist aprepitant. Eur J Cancer 2003;39:1395–401. 12826042 10.1016/s0959-8049(03)00299-5

[16] Erasmus E Mason S van Reenen M. A laboratory approach for characterizing chronic fatigue: what does metabolomics tell us? Metabolomics 2019;15:158. 31776682 10.1007/s11306-019-1620-4

[17] Wolfgram P Zhang L Simpson P. Clinical associations of quarterly Patient Health Questionnaire-9 depression screening results in adolescents with type 1 diabetes. Pediatr Diabetes 2020;21:871–7. 32277561 10.1111/pedi.13017

[18] Gebre BB Deribe B Abeto M. Magnitude and associated factors of depression among hypertensive patients attending treatment follow up in chronic OPD at Hawassa University Comprehensive Specialized Hospital, Hawassa, Southern Ethiopia. Integr Blood Press Control 2020;13:31–9. 32273751 10.2147/IBPC.S240015 PMC7106993

[19] Zhou X Shi H Yang Y. Anxiety and depression in patients with pulmonary arterial hypertension and chronic thromboembolic pulmonary hypertension: results from a Chinese survey. Exp Ther Med 2020;19:3124–32. 32256800 10.3892/etm.2020.8566 PMC7086277

[20] Nieto-Guerrero Gomez JM Silva Vega GP Cacicedo J. Impact of pre-radiation therapy quality of life in lung cancer survival: a prospective, intention-to-treat, multicenter study. Clin Transl Oncol 2020;22:1635–44. 32072471 10.1007/s12094-020-02310-0

[21] Priori R Minniti A Antonazzo B. Sleep quality in patients with primary Sjogren's syndrome. Clin Exp Rheumatol 2016;34:373–9. 27087620

[22] Zhao R Wang Y Zhou W. Associated factors with interstitial lung disease and health-related quality of life in Chinese patients with primary Sjogren's syndrome. Clin Rheumatol 2020;39:483–9. 31578648 10.1007/s10067-019-04753-5

[23] Hao R Dong H Zhang R. The relationship between neuroticism fit and general well-being: the mediating effect of psychological resilience. Front Psychol 2019;10:2219. 31681067 10.3389/fpsyg.2019.02219 PMC6797854

[24] Xiao H Zhang Y Kong D. The Effects of Social Support on Sleep Quality of Medical Staff Treating Patients with Coronavirus Disease 2019 (COVID-19) in January and February 2020 in China. Med Sci Monit 2020;26:e923549. 32132521 10.12659/MSM.923549 PMC7075079

[25] Rogowska AM Zmaczynska-Witek B Mazurkiewicz M. The mediating effect of self-efficacy on the relationship between health locus of control and life satisfaction: a moderator role of movement disability. Disabil Health J 2020;13:100923. 32317244 10.1016/j.dhjo.2020.100923

[26] Mugie SM Benninga MA Di Lorenzo C. Epidemiology of constipation in children and adults: a systematic review. Best Pract Res Clin Gastroenterol 2011;25:03–18. 10.1016/j.bpg.2010.12.010 21382575

[27] Mitani A Jo T Yasunaga H. Venous thromboembolic events in patients with lung cancer treated with cisplatin-based versus carboplatin/nedaplatin-based chemotherapy. Anticancer Drugs 2018;29:560–4. 29570101 10.1097/CAD.0000000000000625

[28] Dore MP Pes GM Bibbo S. Constipation in the elderly from Northern Sardinia is positively associated with depression, malnutrition and female gender. Scand J Gastroenterol 2018;53:797–802. 29779417 10.1080/00365521.2018.1473485

[29] Komiya H Umegaki H Asai A. Prevalence and risk factors of constipation and pollakisuria among older home-care patients. Geriatr Gerontol Int 2019;19:277–81. 30628140 10.1111/ggi.13610

[30] Ghoshal A Salins N Deodhar J. Fatigue and quality of life outcomes of palliative care consultation: a prospective, observational study in a tertiary cancer center. Indian J Palliat Care 2016;22:416–26. 27803563 10.4103/0973-1075.191766 PMC5072233

[31] Sumida K Yamagata K Kovesdy CP. Constipation in CKD. Kidney Int Rep 2020;5:121–34. 32043026 10.1016/j.ekir.2019.11.002 PMC7000799

